# Comparison of Methodologies to Detect Low Levels of Hemolysis in Serum for Accurate Assessment of Serum microRNAs

**DOI:** 10.1371/journal.pone.0153200

**Published:** 2016-04-07

**Authors:** Jaynish S. Shah, Patsy S. Soon, Deborah J. Marsh

**Affiliations:** 1 Hormones and Cancer Group, Kolling Institute of Medical Research, Royal North Shore Hospital, University of Sydney, St. Leonards, New South Wales, Australia; 2 South Western Sydney Clinical School, University of New South Wales, Kensington, New South Wales, Australia; 3 Department of Surgery, Bankstown Hospital, Bankstown, New South Wales, Australia; 4 Medical Oncology Group, Ingham Institute for Applied Medical Research, Liverpool Hospital, Liverpool, New South Wales, Australia; Cleveland Clinic, UNITED STATES

## Abstract

microRNAs have emerged as powerful regulators of many biological processes, and their expression in many cancer tissues has been shown to correlate with clinical parameters such as cancer type and prognosis. Present in a variety of biological fluids, microRNAs have been described as a ‘gold mine’ of potential noninvasive biomarkers. Release of microRNA content of blood cells upon hemolysis dramatically alters the microRNA profile in blood, potentially affecting levels of a significant number of proposed biomarker microRNAs and, consequently, accuracy of serum or plasma-based tests. Several methods to detect low levels of hemolysis have been proposed; however, a direct comparison assessing their sensitivities is currently lacking. In this study, we evaluated the sensitivities of four methods to detect hemolysis in serum (listed in the order of sensitivity): measurement of hemoglobin using a Coulter® AcT diff™ Analyzer, visual inspection, the absorbance of hemoglobin measured by spectrophotometry at 414 nm and the ratio of red blood cell-enriched miR-451a to the reference microRNA miR-23a-3p. The miR ratio detected hemolysis down to approximately 0.001%, whereas the Coulter® AcT diff™ Analyzer was unable to detect hemolysis lower than 1%. The spectrophotometric method could detect down to 0.004% hemolysis, and correlated with the miR ratio. Analysis of hemolysis in a cohort of 86 serum samples from cancer patients and healthy controls showed that 31 of 86 (36%) were predicted by the miR ratio to be hemolyzed, whereas only 8 of these samples (9%) showed visible pink discoloration. Using receiver operator characteristic (ROC) analyses, we identified absorbance cutoffs of 0.072 and 0.3 that could identify samples with low and high levels of hemolysis, respectively. Overall, this study will assist researchers in the selection of appropriate methodologies to test for hemolysis in serum samples prior to quantifying expression of microRNAs.

## Introduction

A class of small non-coding RNAs known as microRNA plays a central role in almost all known biological processes. microRNAs are approximately 17–22 nucleotides in length and when bound to the 3' UTR of target mRNAs, repress gene expression by degradation of target mRNA or suppressing translation [1–3]. The human genome is estimated to encode more than 1,000 microRNAs, which collectively regulate more than half of all protein coding genes [1–4]. Therefore, it is not surprising that aberrant microRNA expression is linked to development and progression of many diseases including cancer [1–3, 5, 6]. Furthermore, microRNA signatures of cancer tissues are associated with cancer types and subtypes as well as staging, progression, prognosis and response to treatments [3, 7–9].

Recently, microRNAs were identified in a range of body fluids including urine, serum, plasma, tears and saliva, highlighting them as potential ‘gold mines’ of noninvasive disease biomarkers [5, 6, 10–14]. Serum microRNAs can withstand extreme conditions such as extended storage, multiple freeze-thaw cycles, high and low pH and even boiling [6, 15, 16]. The encapsulation of microRNA into vesicles (exosomes, microvesicles and high-density lipoproteins), chemical modifications or association with protein complexes such as Ago2, an essential protein for RNA interference, are all currently thought to provide protection against potent endogenous ribonucleases present in the blood [5, 6, 14, 17–20].

The source of microRNAs, collection protocol, extraction and detection methods, as well as inter-individual variables such as age, diet, race and even altitude have been shown to influence the ability to robustly determine microRNA levels. These and other pre-analytical and analytical factors must be addressed in the development of reliable and reproducible microRNA-based tests for clinical settings [15, 16, 21]. In addition, microRNA content released from blood cells upon hemolysis can dramatically alter the expression of certain microRNA, and may lead to false discovery of disease-associated biomarkers [22–24]. One study identified over half of the proposed microRNA biomarkers of solid cancers have been identified at high levels in one or more types of blood cells [25]. Further, up to 65% of detectable microRNAs in plasma have been shown to be affected by hemolysis [23]. While a number of studies have suggested that miR-16 is suitable as a reference microRNA for normalization of samples [26, 27], it is significantly altered by hemolysis, raising some concern for its routine use as a reference microRNA in serum or plasma studies [15, 23].

Currently, there is a lack of consensus on methods to detect low levels of hemolysis in serum that has the potential to affect the accuracy of microRNA-based tests. Reports have revealed that serum microRNA content is already altered due to hemolysis before samples manifest pink discoloration that is visible to the naked eye [23, 24]. In search of methods to detect low levels of hemolysis, Blondal *et al*. (2013) suggested that the ratio of red blood cell-enriched miR-451a to miR-23a, the latter microRNA being unaffected by hemolysis, can be used as a surrogate indicator of hemolysis [22]. miR-451a:miR-23a ratios of <5, 5–7 and >7 are, respectively, indicative of samples at low, moderate or severe risk of hemolysis [22]. The spectrophotometric absorbance of hemoglobin has also been suggested as a measure of hemolysis [22, 24]. A direct comparison of these methods has not been previously reported, nor have their sensitivities to detect low levels of hemolysis in serum been determined.

In this study, we evaluated the sensitivities of four separate methods to detect hemolysis in serum from healthy volunteers and women with ovarian cancer: (1) Coulter^®^ AcT diff™ Analyzer measurement of hemoglobin, (2) visual inspection, (3) spectrophotometric measurement of absorbance of hemoglobin at 414 nm [22–24], and (4) the ratio of miR-451a to miR-23a [22].

## Materials and Methods

### Blood collection, processing and storage

Written consent was obtained and blood collected from 56 women with high-grade serous ovarian carcinoma (HGSOC; 64.1 ± 3.4 years) and 30 healthy females age matched within 5 years (61.0 ± 4.7 years) under a protocol approved by the Northern Sydney Local Health District Human Research Ethics Committee (Protocol #0310-209B). For women undergoing surgical resection of their tumor, blood was collected from the peripherally inserted central catheter (PICC line) prior to the induction of anesthesia. Blood from healthy volunteers was collected using a 21 gauge needle. In both cases, samples were transferred or collected into a 9 ml BD Vacutainer serum tube (BD #455092, North Ryde, NSW, Australia). Blood was allowed to clot for 15–30 minutes at 4°C and centrifuged at 3000 rpm for 15 minutes at 4°C. Serum was carefully withdrawn without disturbing the buffy coat and immediately stored at -80°C in 500 μl aliquots as part of the Kolling Institute Gynecological Tumor Bank. Samples were rapidly thawed in a 37°C water bath as required.

### Hemolysis dilution series

Whole blood, 0.5 ml collected from a healthy volunteer, was allowed to clot in an eppendorf tube, and sonicated until the sample was completely fluid and bright red, indicative of a high degree of hemolysis. Serum was isolated from the blood of a healthy volunteer as per the healthy volunteers’ protocol described above. This sample, collected under optimal conditions, was classified as unhemolyzed for the purpose of this study. A hemolysis dilution series comprising 100%, 20%, 4%, 1%, 0.25%, 0.062%, 0.016%, 0.004%, 0.001% hemolyzed and unhaemolysed samples (v/v) was prepared by serial dilution of the 100% hemolyzed sample with unhemolyzed serum.

### Assessment of hemolysis

Hemolysis in serum samples was measured using 4 methods. The first method measured hemogloblin concentration using the Coulter® AcT diff™ Analyzer and the Tainer Reagent Kit (Beckman Coulter Inc # 8547135, Lane Cove, NSW, Australia). Three technical replicates were performed for each sample. The second method to assess hemolysis was simple visual inspection of serum samples for pink discoloration indicative of free hemoglobin against a white background. Visual discoloration of each sample was scored from 0 (unhemolyzed serum) to 5 (100% hemolyzed serum). The third method was measurement of the absorbance of hemoglobin at 414 nm using a NanoDrop™ 1000 spectrophotometer (Thermo Scientific, Scoresby, Victoria, Australia), averaging two technical replicates per sample. Lastly, the fourth method used determined the ratio of miR-451a to miR-23a-3p (delta Cq (miR-23a-3p—miR-451a)) referred to hereon as the “miR ratio”), with real-time reverse transcription (RT)-PCR using a 7900HT Fast Real-Time PCR System (Life Technologies, Scoresby, Victoria, Australia). Three RT reactions were performed for each sample followed by one PCR per RT.

### RNA extraction and real-time RT-PCR

RNA was extracted from 200 μl serum using the miRCURY RNA isolation kit for Biofluids (Exiqon #300112, Vedbaek, Denmark) according to the manufacturer’s instructions. One μg MS2 bacteriophage carrier RNA (Roche #10165948001, Castle Hill, NSW, Australia) was added during the lysis step. RNA was eluted in 25 μl nuclease-free water twice (final volume 50 μl) and stored at -80°C. RT was performed using 2 μl RNA template per 10 μl RT reaction using the Universal cDNA Synthesis kit (Exiqon #203301). Three independent RT reactions were performed per sample, and one PCR conducted for each. The real-time RT-PCR master mix contained 100-fold diluted cDNA, 1X ExiLENT SYBR Green master mix (Exiqon #203420) and 1X ROX reference dye (Life Technologies #12223–012). Ten μl of the master mix was transferred to each well of a Serum/Plasma Focus microRNA PCR panel (Exiqon #203843) or Pick-&-Mix microRNA PCR panel (Exiqon #203802) containing locked nucleic acid (LNA) primers using the epMotion 5070 liquid handling system (Eppendorf, North Ryde, NSW, Australia). Real-time RT-PCR was performed on a 7900HT Fast Real-Time PCR System (Life Technologies). PCR conditions were as follows: 95°C for 10 minutes; followed by 40 amplification cycles of 95°C for 10 seconds and 60°C for 1 minute, followed by melting curve analysis.

### Statistical analyses

Real-time RT-PCR data was exported and analyzed in GenEx software V6 (Exiqon #207016) and adjusted for plate-to-plate PCR variability using the spike-in miR UniSP3. Further analyses were performed in the statistical language R using ‘ggplot2’, ‘ROCR’, ‘verification’, ‘car’ and ‘plotrix’ packages. An average of the technical replicates was calculated for each sample. A *P-value* of <0.05 using the student’s t-test assuming equal variance or Mann–Whitney *U* test was considered as significant. Results are presented as mean ± 2 × S.E.M. Error bars in all Figs represent S.E.M.

## Results

### Sensitivities of four methods to detect hemolysis

**Fig 1:**
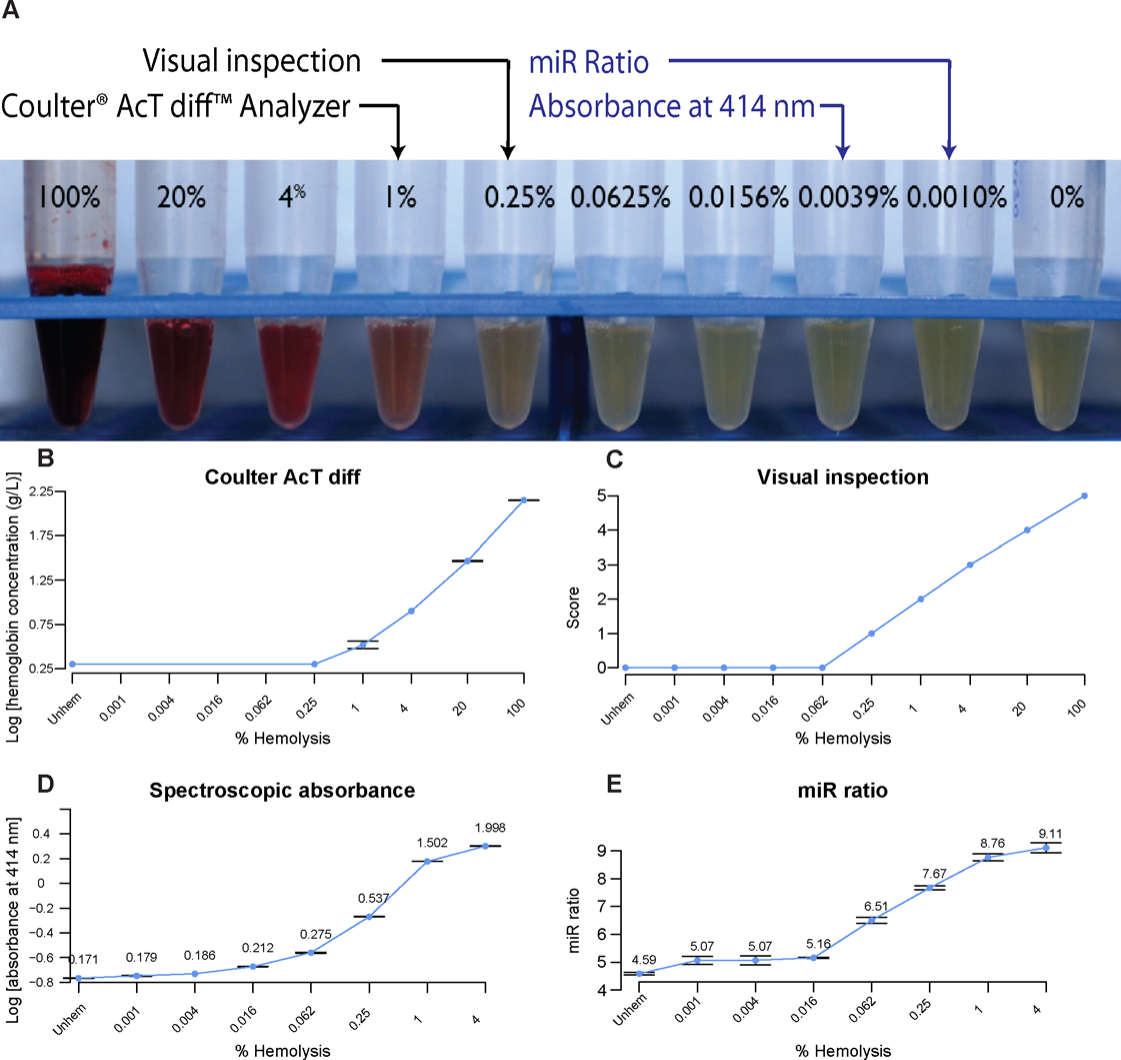
Sensitivities of four methods to detect hemolysis. **(A)** A hemolysis series was prepared by diluting 100% hemolyzed sample with unhemolyzed serum (0%), and the sensitivity of each method determined by its ability to detect hemolysis (indicated by arrows). **(B—E)** Detection of hemolysis using four methods. For visual inspection, samples were scored from 0 (unhemolyzed sample) to 5 (100% hemolysis). Averages of technical replicates are shown where appropriate. ‘Unhem’ denotes unhemolyzed serum. Absorbance measures (D) and miR ratios (E) are noted on the graphs.

Four methods for the detection of hemolysis were compared using the hemolysis dilution series as described. The Coulter^®^ AcT diff™ Analyzer measurement of hemoglobin could detect down to 1% hemolysis, while the 0.25% hemolyzed and the unhemolyzed sample remained indistinguishable (Fig 1B). By visual inspection alone, the pink discoloration of free-hemoglobin could be detected down to 0.25% hemolysis (Fig 1A and 1C; S1 File). In contrast, the spectrophotometric method could detect down to 0.004% hemolysis (Fig 1D). The calculated miR ratio could detect down to 0.001% hemolysis, the lowest point tested, making it the most sensitive method (Fig 1E). The Coulter^®^ AcT diff™ method was excluded from further analyses due to its low sensitivity. Therefore, in order of decreasing sensitivity, the methods used can be ranked as miR ratio > spectrophotometry > visual inspection > Coulter^®^ AcT diff™ Analyzer.

**Fig 2:**
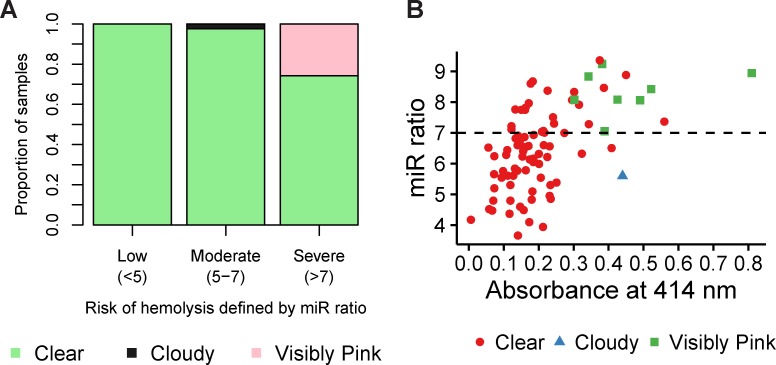
Comparison of methodologies for determining hemolysis in serum samples. Serum samples (N = 86) categorized by low (miR ratio <5; N = 14), moderate (miR ratio 5–7; N = 41) and severe (miR ratio >7; N = 31) hemolysis. Results of visual inspection are recorded for each category as the proportion of samples that are clear, cloudy or visibly pink. **(B)** Absorbance at 414 nm and the miR ratio of the cohort (N = 86). The dotted line represents the threshold above which samples are considered to be severely hemolysed according to the miR ratio (>7). Samples are color-coded according to their visual appearance (clear, cloudy or visibly pink).

Next, we determined the hemolysis levels of 86 samples (56 women with ovarian cancer and 30 age-matched, healthy females) using visual inspection, spectrophotometric absorbance and the miR ratio (S2 File). Using the miR ratio as the ‘gold standard’, 16% (14/86), 48% (41/86) and 36% (31/86) of the samples were found to have low (miR ratio <5), moderate (miR ratio between 5 and 7) or severe (miR ratio >7) hemolysis, respectively, according to the criteria defined by Blondal *et al*. (2013), highlighting hemolysis as potentially a problematical factor, even when serum samples are collected under optimal conditions (Fig 2A). The miR ratio of the unhemolyzed sample used to construct the dilution series was 4.59 ± 0.10. In contrast, the miR ratio for the 0.25% hemolyzed sample, the limit of visual inspection, was 7.67 ± 0.14. Furthermore, 100% of the samples with pink discoloration (8/8) had a miR ratio >7, suggesting that any samples with visible pink discoloration were already severely affected by hemolysis (Fig 2A).

### Identification of severely hemolyzed samples using visual inspection and the absorbance of hemoglobin

Since the majority of the severely hemolyzed samples based on the miR ratio (74%; 23/31) were visually undetectable, we investigated whether the absorbance of hemoglobin could be utilized to detect additional severely hemolyzed samples, i.e miR ratio >7. The samples with pink discoloration had both higher absorbance (0.46 ± 0.11) and miR ratios (8.29 ± 0.51; Fig 2B).

**Fig 3:**
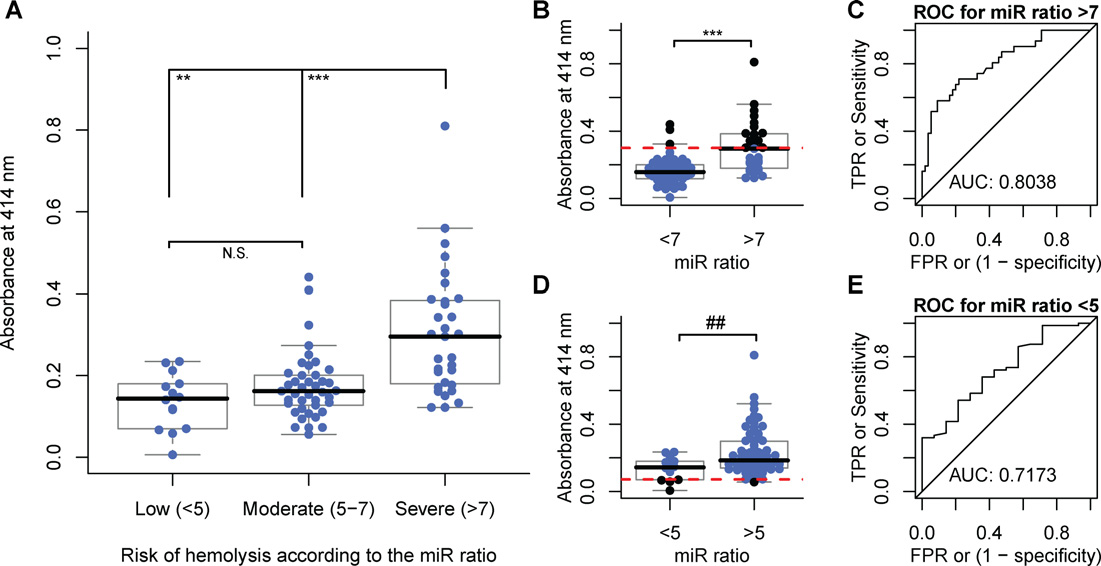
Identification of samples with low or severe hemolysis by spectrophotometric absorbance. **(A)** Cohort (N = 86) is grouped by low (miR ratio <5; N = 14), moderate (miR ratio 5–7; N = 41) and severe (miR ratio >7; N = 31) predicted risk of hemolysis, and absorbance at 414 nm was compared between groups. No significant differences in absorbance of samples were observed between the low and moderate groups; however, both were significantly different to the severe hemolysis group. **(B-C)** Absorbance of samples with miR ratio >7 was 1.85-fold higher than those with miR ratio <7. ROC analysis suggested that absorbance could predict severely hemolyzed samples (miR ratio >7). The cut-off for absorbance of 0.3 identified by ROC is shown as a dotted red line. **(D-E)** ROC analysis revealed a cut-off for absorbance of 0.072 (depicted as a dotted red line) below which samples would be predicted to have low levels of hemolysis (miR ratio <5). ** *P* < 0.001, *** *P* < 0.0001 and ## Mann-Whitney *U* test *P* < 0.001. ‘TPR’ and ‘FPR’ refer to true and false positive rates, respectively.

No significant differences were observed between the absorbance of low and moderate hemolyzed samples as classified by the miR ratio (*P* = 0.13; Fig 3A); however, low and moderately hemolyzed samples were significantly different from the severely hemolyzed samples (*P* <0.001 & <0.0001, respectively). In other words, the miR ratio could further quantify hemolysis in the samples that were indistinguishable by absorbance. Severely hemolyzed samples (miR ratio >7) had 1.85-fold higher absorbance than low and moderately hemolyzed samples combined together (miR ratio <7; *P* <0.0001; Fig 3B), indicating that the absorbance of hemoglobin could have predictive value in discriminating severely hemolyzed (miR ratio >7) samples. The receiver operator characteristic (ROC) curve separated severely hemolyzed samples from the rest with an area under curve (AUC) of 0.8038 (*P* <0.0001; Fig 3C). The cut-off absorbance of 0.3 identified 48.4% (15/31) of hemolyzed samples, of which 8 were visually undetectable. The accuracy of prediction using this cut-off ($\frac{True\;Positive+True\;Negative}{Positive+Negtive}$) was 0.779.

We tested whether a similar analysis could identify an absorbance cut-off below which a sample is likely to have low levels of hemolysis (miR ratio <5; Fig 3D). Despite similar median values between the two groups (miR ratio <5 and >5), the cut-off absorbance of 0.072 could identify samples with a low risk of hemolysis with AUC of 0.7173 (Fig 3E). In general, absorbance-based tests to predict hemolysis suffered from low sensitivity, but offered high specificity and moderate positive and negative predictive values (Table 1).

**Table 1:** Assessment of performance of the spectrophotometric absorbance of hemoglobin at 414 nm for predicting the miR ratio.

	Low risk (miR ratio <5)		Severe risk (miR ratio >7)	
	All samples (N = 86)	Clear samples* (N = 77)	All samples (N = 86)	Clear samples* (N = 77)
**Cut-off absorbance**	0.072	0.072	0.300	0.300
**AUC**	0.717	0.733	0.804	0.756
**Accuracy**	0.849	0.831	0.779	0.779
**Sensitivity**	0.250	0.250	0.484	0.333
**Specificity**	0.986	0.984	0.946	0.981
**PPV**	0.800	0.800	0.833	0.889
**NPV**	0.852	0.833	0.765	0.765

Absorbance measurements of samples were split into two groups for each comparison: (I) prediction of low risk of hemolysis (miR ratio <5): <5 versus >5, and (II) prediction of high risk of hemolysis (miR ratio >7): >7 versus <7. ROC analysis was performed for each comparison, and the cut-off absorbance that maximized the accuracy of prediction was selected. Sensitivity, specificity, PPV and NPV were calculated based on the chosen cut-off.

PPV: positive predictive values

NPV negative predictive values

*refers to samples that did not have a pink discoloration or were cloudy.

### Impact of hemolysis on hemolysis-sensitive microRNAs

**Fig 4:**
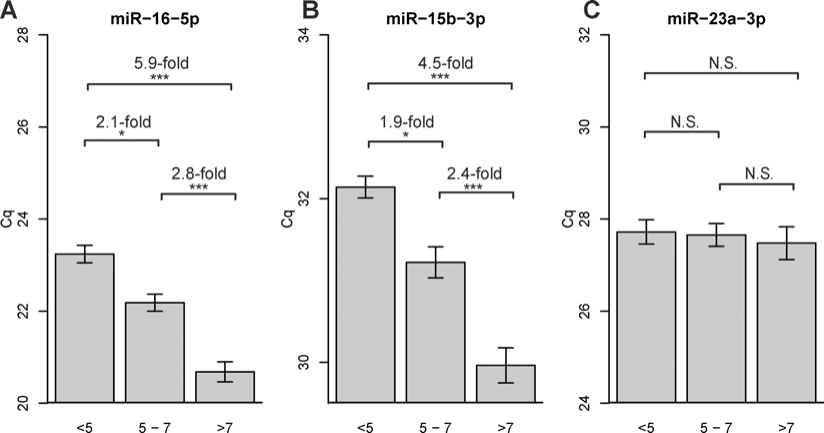
Hemolysis-sensitive high and low abundant microRNAs are significantly altered between categories defined by the miR ratio. **(A)** Levels of hemolysis-sensitive highly abundant serum microRNA miR−16−5p was found to be significantly altered across low, moderate and severely hemolyzed serum samples defined by miR ratios **(B)** Levels of a hemolysis-sensitive low abundant microRNA miR−15b−3p were also different across all miR ratio categories. **(C)** miR−23a−3p was present at a similar level amongst three categories, supporting its use as a reference microRNA in determining the miR ratio. * *P* <0.05, ** *P* < 0.001 and *** *P* < 0.0001.

Since the microRNAs affected by hemolysis originate predominantly from the rupture of red blood cells (RBC), the extent to which a specific microRNA is altered may depend on its abundance in RBC. Using RBC-derived miR-16-5p and miR-15b-3p surrogates for hemolysis-sensitive high and low abundant microRNAs based on Cq values in the cohort as a whole (Fig 4), we calculated differences in their levels across the 3 categories defined by the miR ratio, especially for miR-15b-3p as most microRNAs in serum are likely to present at moderate or low levels. The levels of miR-16-5p and miR-15b-3p increased as the miR ratio increased (Fig 4A and 4B). miR-16-5p and miR-15b-3p were found to be altered by 5.9-fold (*P* <0.0001) and 4.5-fold (*P* <0.0001), respectively, in the samples at severe risk of hemolysis (miR ratio >7) compared to those at low risk (miR ratio <5). Both microRNAs were also found to be elevated by approximately two-fold between miR ratio categories <5 compared to 5–7, as well as 5–7 compared to >7. Thus, both high and low abundant microRNAs susceptible to hemolysis are significantly altered amongst 3 categories defined by the miR ratio. miR-23a-3p was present at a similar level in each of the 3 categories as expected (Fig 4C).

## Discussion

Serum microRNAs are attractive non-invasive biomarkers because of their disease-specific expression and stability in a wide range of conditions. However, a series of pre-analytical and analytical variables must be considered in the development of robust and reliable microRNA-based tests [1, 2, 5–7, 9, 15, 16, 21]. The effect of release of the microRNA content of blood cells upon hemolysis dramatically alters the level of specific serum microRNAs. Recent studies have shown that 58% (46/79) of proposed microRNA biomarkers [25] for solid cancers were highly expressed in one or more blood cell types, and up to 65% of detectable microRNAs in plasma were affected by hemolysis [23]. Hemolysis in clinical samples is common. Reports have suggested that approximately 43% of clinical samples are hemolyzed as determined by free hemoglobin >0.5 g/L, whereas visual detection indicated by the presence of a pink discoloration is seen in less than 6% of samples [28, 29]. Therefore, quantifying hemolysis is an essential step for any procedure measuring circulatory microRNAs for diagnostic purposes or biomarker discovery. A number of methods to measure hemolysis in serum have been described; however, a direct comparison assessing their sensitivities has not been reported.

In our study of serum, using a 4-fold dilution series, visual inspection could only detect down to 0.25% hemolysis (v/v). This is comparable to the detection limit of 0.125% (v/v) identified by Kirschner *et al*. (2011) using a 2-fold dilution series of plasma [24]. We and others have shown that visual inspection, i.e. identification of visible pink, as a measure of hemolysis is insufficient as levels of hemolysis-sensitive microRNA such as miR-451a are already compromised prior to visual detection [22, 24].

The ratio of miR-451a to miR-23a-3p was found to be the most sensitive method that could detect down to 0.001% hemolysis in serum. We quantified hemolysis levels of 86 samples using the miR ratio, and discovered that hemolysis-sensitive microRNAs miR-15b-3p and miR-16-5p were significantly affected in the categories of hemolysis (low, moderate and severe) defined by Blondal *et al*. (2013) [22]. In particular, the levels of low abundant, hemolysis-sensitive miR-15b-3p were approximately 4.5-fold higher in the samples with low (miR ratio <5) versus severe (miR ratio >7) risk of hemolysis. The differences were greater for the more abundant miR-16-5p between the same groups (5.9-fold).

**Fig 5:**
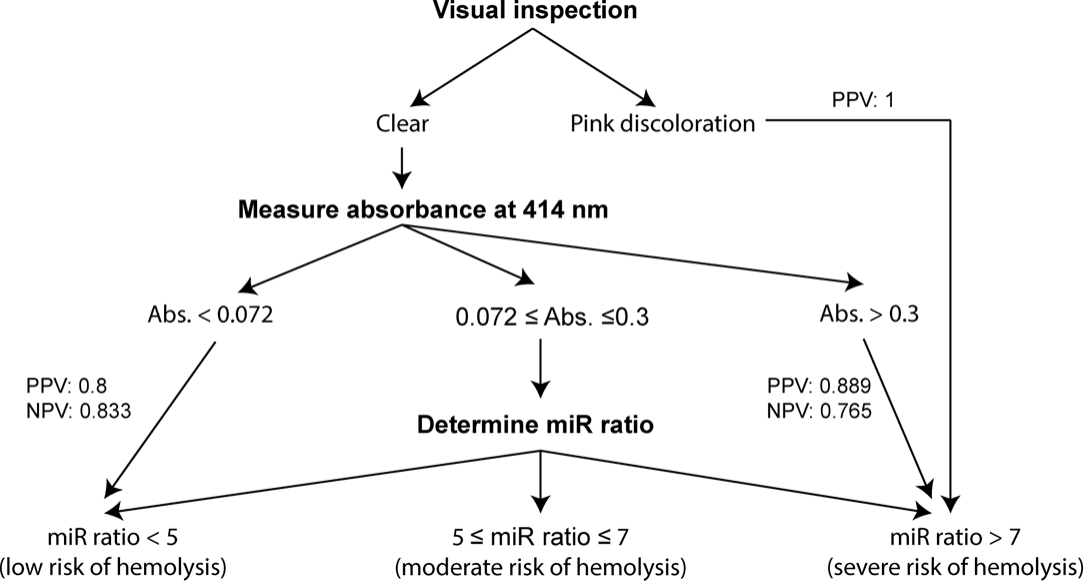
Assessment of hemolysis in serum samples. All serum samples exhibiting pink discoloration were found to be strongly affected by hemolysis for microRNA profiling according to the miR ratio. After exclusion of the visibly hemolyzed samples, samples with absorbance at 414 nm of >0.3 are also likely to be have miR ratio >7, predicting severe hemolysis. In contrast, samples with an absorbance at 414 nm of <0.072 are predicted to have a miR ratio <5. Samples meeting these criteria may be excluded from miR ratio for the purpose of determining hemolysis; however, the miR ratio should be determined for samples with absorbance between 0.072 and 0.3. PPV and NPV refer to positive and negative predictive values after removal of visibly hemolyzed or cloudy samples, respectively.

While the miR ratio was the most sensitive method to detect low levels of hemolysis, it may not be suitable for all large-scale screening for hemolysis due to additional cost and a relatively large requirement for starting material (200 μl serum). The absorbance of hemoglobin at 414 nm, on the other hand, is a suitable alternative as it overcomes these restrictions and was found to be more sensitive than visual inspection in our dataset. We tested whether absorbance could identify samples that are at a severe risk of hemolysis (miR ratio >7) but remained undetectable by visual inspection. ROC analysis revealed that absorbance at 414 nm >0.3 (water as blank) identified severely hemolyzed samples with accuracy, PPV and NPV of approximately 80% (Fig 5). Over half (8/15) of the samples were visually undetectable. Similarly, Kirschner *et al*. (2013) suggested use of absorbance at 414 nm >0.2 (unhemolyzed plasma as blank) to identify hemolysis in plasma as it reduced variability in miR-451 levels [23]. The different choices of blanks and serum versus plasma may have led to the differences observed. Interestingly, some samples in our study had lower absorbance than the unhemolyzed serum used in the dilution series; therefore, water seemed to be an appropriate choice of blank. Similarly, based on our data, samples with absorbance less than 0.072 are likely to be at low risk of hemolysis (miR ratio <5).

Given that a substantial number of circulatory microRNAs are known to be affected by hemolysis, the miR ratio is recommended as the final quality control step unless clearly indicated in the literature that the microRNA of interest is not affected by hemolysis, for example miR-122 [15, 30, 31]. If a promising microRNA is found to be sensitive to hemolysis, clinical interpretation of the test should evaluate the extent to which it is modified by the underlying condition or disease as well as hemolysis. Since hemolysis is common in clinical samples, this comparison will also help identify levels of hemolysis that are acceptable in the samples without significantly affecting the performance of a given test. Failure to meet quality standards would jeopardize accuracy of measurement in the context of a disease specific relationship of any microRNA known to be affected by hemolysis. Measuring absorbance of hemoglobin at 414 nm can identify samples that are likely to be at either a low or severe risk of hemolysis, reducing the total number of samples that would require testing by miR ratio to determine hemolysis. Despite high specificity, we have shown that the absorbance-based method is inaccurate for predicting hemolysis between absorbance readings at 414 nm of 0.3 and 0.072, and suggest that the miR ratio should be used to test for hemolysis in samples that fall within this range. Further, bilirubin is known to interfere with the absorbance of hemoglobin, rendering this method inaccurate in conditions such as jaundice where serum bilirubin levels are elevated [32].

## Conclusion

The pivotal role of hemolysis as a quality control measure in any serum microRNA profiling cannot be underestimated. The ratio of miR-451a to miR-23a-3p proposed by Blondal *et al*. (2013) was found to be the most sensitive method to detect low levels of hemolysis, and should be routinely employed. High and low abundant microRNAs sensitive to hemolysis are significantly altered in the three categories of hemolysis (low, moderate and severe) defined by Blondal *et al*. (2013). Visual inspection to detect hemolysis is insufficient as microRNA in serum samples that do not display a visible pink discoloration can still show effects of hemolysis, as shown by a miR ratio >7. Measuring hemoglobin’s absorbance at 414 nm can identify samples that are likely to be at a low or high risk of hemolysis, therefore reducing the total number of samples that should be further analysed for hemolysis using the miR ratio test.

## Supporting Information

S1 File Sensitivities of four methods to detect hemolysis. This table shows levels of hemolysis measured by Coulter® AcT diff™ Analyzer, visual inspection, absorbance at 414 nm and the miR ratio in the hemolysis dilution series. The values represent averages of technical replicates.
(XLSX)

S2 File Hemolysis levels in the cohort. This table shows hemolysis levels in 86 serum samples measured by visual inspection, absorbance at 414 nm and the miR ratio. In addition, levels of hsa-miR-16-5p and hsa-miR-15b-3p are also displayed. The values represent averages of technical replicates.
(XLSX)
